# Effects of pelleted versus loose salt-limited protein supplement on supplement intake behavior of yearling heifers grazing dryland pastures

**DOI:** 10.1093/tas/txac115

**Published:** 2022-08-23

**Authors:** Hayley C White, Megan L Van Emon, Hannah M DelCurto-Wyffels, Samuel A Wyffels, Timothy DelCurto

**Affiliations:** Department of Animal and Range Sciences, Montana State University, PO Box 172900, Bozeman, MT 59717-2900, USA; Department of Animal and Range Sciences, Montana State University, PO Box 172900, Bozeman, MT 59717-2900, USA; Department of Animal and Range Sciences, Montana State University, PO Box 172900, Bozeman, MT 59717-2900, USA; Department of Animal and Range Sciences, Montana State University, PO Box 172900, Bozeman, MT 59717-2900, USA; Department of Animal and Range Sciences, Montana State University, PO Box 172900, Bozeman, MT 59717-2900, USA

**Keywords:** physical form of supplement, salt-limited supplement, supplement intake behavior, yearling heifers

## Abstract

The objectives of this study were to evaluate the impacts of supplement form on supplement intake behavior, body weight (BW), and body condition score (BCS) change of yearling heifers grazing dryland pastures during the summer. In each of the two years, Angus crossbred heifers (14 mo of age; year 1, *N* = 57, BW = 449 ± 3.60 kg; year 2, *N* = 58, BW = 328 ± 3.57 kg) were used in a 84-d completely randomized design evaluating the following treatments: 1) control, no supplement; 2) salt-limited supplement in pelleted form; and 3) a salt-limited supplement in loose form. Individual supplement intake, and time spent at the feeder were measured throughout the course of the study using a SmartFeed Pro self-feeder system (C-Lock Inc., Rapid City, SD, USA). On days 0, 42, and 84, the heifers were weighed, and body condition scored following a 16-h shrink. Supplementation and form of supplement did not influence (*P* ≥ 0.62) BW change for yearling heifers within or across study grazing periods. Body condition score was not influenced (*P* ≥ 0.26) by supplementation and form within the 0 to 42 (period 1) or 42 to 84 (period 2)-d periods but displayed a treatment by year interaction (*P* < 0.01) for the 84-d summer grazing period. Supplement intake (kg/d and g/kg BW) displayed a treatment × period interaction (*P* < 0.01). Supplement intake (kg/d) of heifers consuming pelleted supplement was 28% and 31% greater (*P* ≤ 0.02) than heifers consuming loose supplement in periods 1 and 2, respectively. Supplement intake (g/kg BW) of heifers consuming pelleted supplement was 24% and 32% greater (*P* ≤ 0.05) than heifers consuming loose supplement in periods 1 and 2, respectively. Overall, across both years, supplement intake in period 1 was less than half (*P* < 0.01) that of period 2, averaging 0.50 and 1.14 kg/day, respectively. Variation in supplement intake (% CV) was greater (*P* = 0.03) in period 1 compared to period 2, averaging 119% and 91%, respectively. In addition, variation in supplement intake was greater (*P* = 0.03) in year 2 than year 1, averaging 122% and 88%. Our results suggest that salt-limited supplements have a high degree of intake variation and pelleting could have a masking effect as indicated by the greater intake and intake rate of supplement with heifers consuming the pelleted supplement.

## Teaser

Self-fed salt-limited supplements have a high degree of intake variation. Pelleted salt-limited supplement resulted in greater intake and intake rate compared to non-pelleted supplement.

## INTRODUCTION

Western beef producers often graze cattle on arid and high elevation rangelands where seasonal deficiencies of nutrients are frequent (DelCurto et al., 2000). To offset seasonal deficiencies of nutrients, protein supplements are used to increase forage intake and improve animal performance (McCollum and Horn, 1990; Bowman and Sowell, 1997; Bodine et al., 2001). Therefore, forage-based production systems must develop strategies that maximize forage use while minimizing supplemental inputs in order to reduce feed costs and maintain acceptable levels of beef cattle performance (Bowman et al., 1995; DelCurto et al., 2000). The strategy, or goal of strategic supplementation, should be to use the most efficient feed delivery system to minimize costs and utilize supplements that reduce variation of animal intake (Bowman and Sowell, 1997; DelCurto et al., 2000; Kunkle et al., 2000). Multiple supplement delivery systems and forms are available commercially to meet animal nutrient demands including loose meal, liquid, pellets, cubes, and blocks, which can be either hand-fed or self-fed (Bowman et al., 1995; Bowman and Sowell, 1997).

Under most rangeland cattle production scenarios, self-fed systems are often preferred due to ease of delivery and reduction in labor. However, self-fed supplementation programs assume that animals, when group fed, consume a targeted quantity of supplement (Bowman and Sowell, 1997; DelCurto et al., 2000). This assumption does not consider variation in intake by individual animals and the potential negative outcomes on animal performance and/or decreased profit margins for the producer if supplement is not consumed at the targeted amount (Bowman and Sowell, 1997; Williams et al., 2018; Wyffels et al., 2020).

In addition to delivery method, Bowman and Sowell (1997) suggest that there are other factors that affect variation of supplement intake, such as supplement form. Loose (finely ground) supplement and pelleted supplement are two popular formulations for protein supplements. The most common method to limit intake of self-fed supplements is the use of salt ranging from 20% to 30% of the supplement composition (Weir and Torell, 1953; Kunkle et al., 2000). However, it has been proposed that supplement form can mediate the effectiveness of salt as an intake limiter for self-fed supplement (Hentges et al., 1967; Dove and Freer, 1986; Kunkle et al., 2000).

Research evaluating the effectiveness of salt as an intake limiter within different supplements forms (loose and pelleted) and the effect of supplement form on supplement intake behavior is limited. Therefore, our research evaluated the impacts of supplement form (loose vs. pelleted) on individual supplement intake and intake behavior of yearling heifers consuming a self-fed, salt limited supplement while grazing low-quality forages. We hypothesized that pelleting of supplement will have a masking effect on salt, resulting in increased supplement intake and influence intake behavior.

## MATERIALS AND METHODS

Experimental procedures described herein were approved by the Agriculture Animal Care and Use Committees of Montana State University (#2017-AA09). All animals used in this study were provided by the Montana Agricultural Experiment Station. This study was conducted at the Fort Ellis Research Center (45°39ʹ16″N, 110°58ʹ35″W) at Montana State University in Bozeman, Montana, USA. The average precipitation is 46.9 cm with snow representing 59.3%. The average temperature is 9.74°C with 113 total growing season days.

This study was conducted with Angus crossbred heifers (14 mo of age) summer grazing a 93-ha dryland pasture. Heifers were stratified by body condition score (BCS) and body weight (BW; *N* = 57 heifers in year 1, average BW = 449 ± 3.60 kg; *N* = 58 heifers in year 2, average BW = 328 ± 3.57 kg) and, within stratum, randomly allotted to one of three supplement treatments: 1) control, no supplement; 2) 25% salt-limited supplement in pelleted form (approximately 5-mm diameter); and 3) 25% salt-limited supplement in loose form (finely ground and mixed). The pelleted and loose forms of the supplement were isonitrogenous, isocaloric, and formulated to meet the protein needs of yearling cattle on summer pasture (Table 1). Differences between the supplement composition were primarily due to the addition of binding agents for pelleting. The target daily supplement intake was 0.91 kg/heifer.

**Table 1.** Composition of supplements developed for yearling heifers grazing summer pastures

Ingredient, % DM basis	Loose	Pelleted
Wheat middlings	57.10	53.54
Salt	25.00	25.00
Soybean meal	8.50	9.50
Calcium carbonate	5.50	5.45
Molasses	2.50	5.00
Bentonite powder	1.00	1.00
Dicalcium phosphate	0.15	0.25
Trace mineral package	0.10	0.10
Bovatec 91-Dry^1^	0.07	0.07
Selenium 1600	0.06	0.06
Vitamin package	0.02	0.02
Chemical composition, % DM basis		
Total digestible nutrients	48.68	47.64
Crude protein	14.14	14.09
Acid detergent fiber	6.56	6.23
Neutral detergent fiber	21.09	19.92

Bovatec by Zoetis Services LLC, Parsippany, NJ.

**Figure 1.**
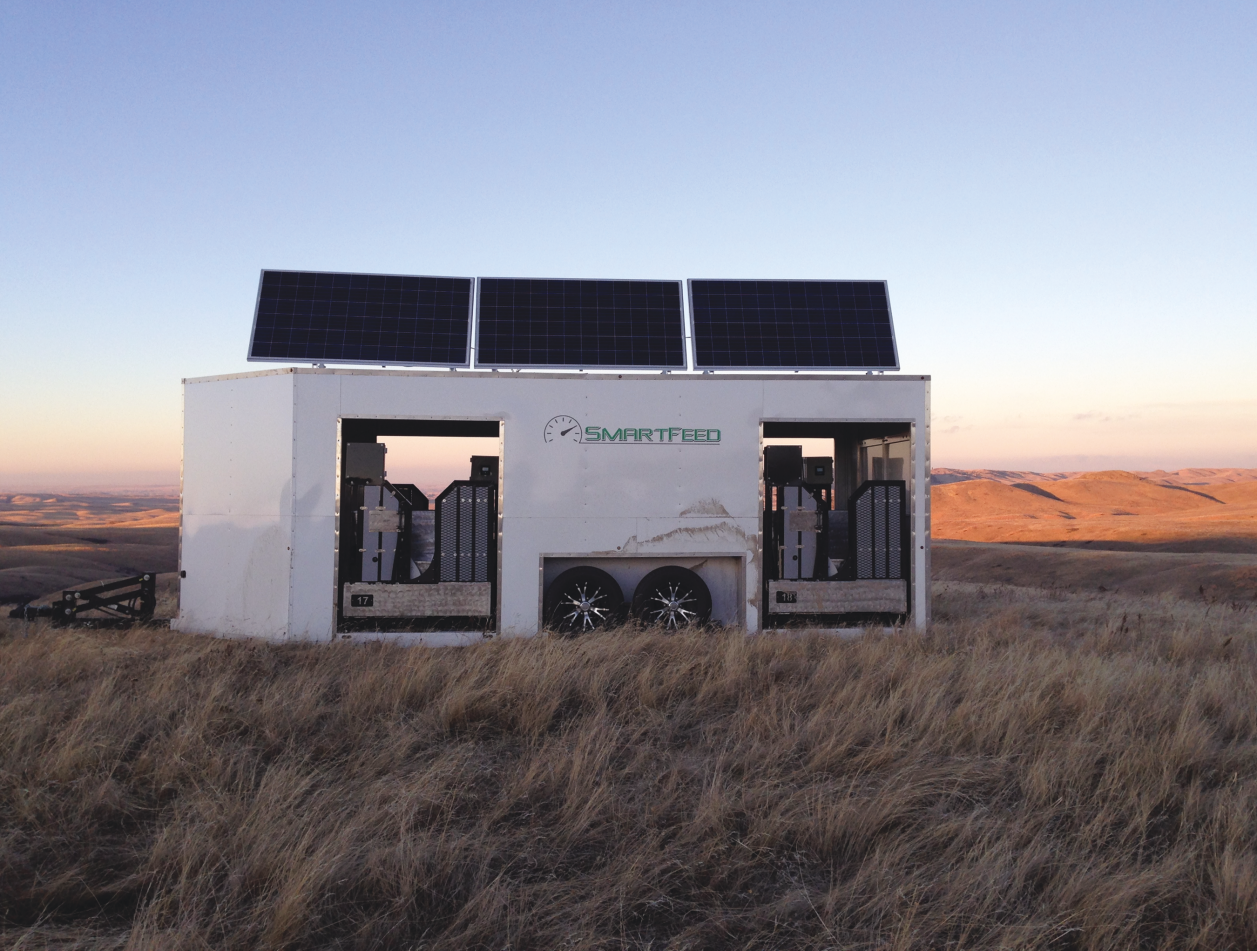
SmartFeed Pro (C-Lock Inc., Rapid City, SD USA) Feed Unit Trailer. Trailer has four feeding stations with two feeders randomly assigned to each treatment supplement.

Each heifer was equipped with an electronic identification tag (Allflex USA, Inc., Dallas-Fort Worth, TX, USA) for the measurement of individual supplement intake (kg/day and g/kg BW/day), time spent at the feeder (minutes), and intake rate (g/min) using a SmartFeed Pro self-feeder system (C-Lock Inc., Rapid City, SD, USA; Figure 1), which provided a total of four individual feeding stations (Wyffels et al., 2020). The SmartFeed Pro unit limits access to the feedbunks via mechanical locking gates which allows multiple treatments within a single pasture. Two feeding units supplied the loose supplement, and two units supplied the pelleted supplement with the control animals locked out of all four units. The SmartFeed Pro trailer was centrally located in the pasture within 500-m of a water source. Treatment supplement feed units were randomly assigned for both the north and south facing directions. By recording individual animal feeding events, we were able to measure and account for supplement consumption by animals not in the assigned treatment. In each year, supplemented heifers consumed 2.89% and 3.86% from the wrong treatment group, for year 1 and 2, respectively. In contrast, control heifers (non-supplemented heifers), consumed 11.78% and 9.58% of the total supplement intake in year 1 and 2, respectively, which corresponded to mechanical failures of the gate locking mechanisms on the SmartFeed Pro system.

BW and BCS were collected on days 0, 42, and 84 following a 16-hr shrink. Study periods consisted of 42 d each with period 1 on days 0 to 42 and period 2 on days 43 to 84. Heifer BCS was evaluated independently by two observers using a 9-point scale (1 = extremely emaciated, 9 = extremely obese; Neumann and Lusby, 1986). The same technicians measured BCS throughout the study. Pasture production was measured by clipping a 0.25 m^2^ plot at 10 sites on days 0, 42, and 84 (Table 2). All clipped samples were composited by time period and sent to a commercial laboratory (Dairy One, Ithaca, NY) and analyzed for DM, CP, TDN, NDF, and ADF.

**Table 2.** Forage production (kg/ha) and composition (%) of improved summer pastures grazed by yearling heifers during summer grazing period (84 d) over 2 yr in Bozeman, MT, USA

	Production	DM	TDN	CP	NDF	ADF
Year 1						
Day 42	1915	93.7	61	8.9	57.7	35.1
Day 84	719	93.3	59	5.3	65.2	42.1
Year 2						
Day 0	2181	92.3	61	9.9	57.5	36.1
Day 42	1082	94.7	57	5.8	72.1	45.4
Day 84	659	94.9	60	5.9	60.8	37.2

The effects of supplement form on daily supplement intake, time spent at the supplement feeder, and the rate of supplement intake were analyzed using generalized linear mixed models in an ANOVA framework with supplement treatment, period, year, and all two-way interactions as fixed effects and individual animal as a random effect. The effects of supplementation, supplement form on heifer BW and condition change within the 42-d grazing periods and across the total 84-d summer grazing period were analyzed using ANOVA with generalized linear models for a complete randomized design with treatment, year and their interaction as fixed effects. Data were plotted and log-transformed if needed to satisfy assumptions of normality and homogeneity of variance. Statistical significance was accepted at an alpha of <0.05. All statistical analyses were performed in R (R Core Team, 2017).

## RESULTS

Influence of supplementation and form on performance variables are listed in Table 3. There was a year effect (*P* < 0.01) on initial heifer BW with heifers in year 2 being 36.9% lighter than heifers in year 1. Likewise, heifer BCS was 0.68 units lower (*P* < 0.01) in year 2 compared to heifers in year 1. Supplementation and form of supplement did not influence (*P* ≥ 0.62) body weight change for yearling heifers within or across study grazing periods. Body condition was not influenced (*P* ≥ 0.26) by supplementation and form within periods 1 and 2, but displayed a treatment by year interaction (*P* < 0.01) for the 84-d summer grazing period where year 1 heifers provided loose supplement had greater BCS gains than non-supplemented heifers (*P* = 0.02) and heifers provided pelleted supplement (*P* = 0.03), and no treatment effects were observed in year 2 (*P* ≥ 0.18). Over the 84-d period, BW gains were reduced (*P* < 0.01) in year 1 compared to year 2, averaging 0.78 kg/d and 1.17 kg/d, respectively.

**Table 3.** Influence of supplementation and form of supplement on yearling heifer performance over two summers grazing improved dryland pastures

Item	Treatments^1^			SEM^2^	*P* – values		
	Control	Loose	Pelleted		TRT^3^	YR^4^	TRT×YR^5^
Initial							
Body weight, kg					0.99	<0.01	0.88
Year 1	449.0	449.0	449.0	6.23			
Year 2	330.0	324.0	329.0	6.18			
Body condition					0.88	<0.01	0.93
Year 1	5.14	5.11	5.14	0.06			
Year 2	4.49	4.40	4.47	0.06			
Period 1, 0 to 42 d							
Δ Body weight, kg	45.80	43.80	42.40	1.44	0.62	0.20	0.62
Δ Body condition					0.26	<0.01	0.85
Year 1	0.13	0.29	0.17	0.07			
Year 2	0.50	0.61	0.46	0.07			
Period 2, 42 to 84 d							
Δ Body weight, kg					0.84	<0.01	0.21
Year 1	23.0	24.7	23.0	2.28			
Year 2	49.1	54.0	57.1	2.22			
Δ Body condition					0.34	0.03	0.01
Year 1	0.39	0.52	0.37	0.08			
Year 2	0.15	-0.75	0.25	0.08			
0 to 84 d							
Δ Body weight, kg					0.69	<0.01	0.42
Year 1	67.0	67.7	64.7	2.58			
Year 2	96.8	97.7	100.4	2.57			
Δ Body condition					<0.01	0.22	<0.01
Year 1	0.53	0.79	0.54	0.07			
Year 2	0.65	0.54	0.71	0.07			

Treatments are 1) Control, no supplement, 2) Supplement in loose form, 3) Supplement in pelleted form.

SEM = Standard Error (*N* = 20).

Treatment main effect (TRT).

Year main effect (YR).

Treatment by year interaction.

Influence of physical form of supplement on supplement intake behavior variables are listed in Table 4. Supplement intake (kg/d and g/kg BW) displayed a treatment × period interaction (*P* < 0.01). Supplement intake (kg/d) of heifers consuming pelleted supplement was 28% and 31% greater (*P* ≤ 0.02) than heifers consuming loose supplement in periods 1 and 2, respectively. Supplement intake (g/kg BW) of heifers consuming pelleted supplement was 24% and 32% greater (*P* ≤ 0.05) than heifers consuming loose supplement in periods 1 and 2, respectively. Additionally, supplement intake (g/kg BW) displayed a year effect (*P* < 0.01), where heifers consumed 0.55 g/kg BW more in year 2 than in year 1 (1.75 ± 0.13 vs. 2.30 ± 0.13 g/kg BW). Overall, across both years supplement intake in period 1 was less than half (*P* < 0.01) that of period 2, averaging 0.50 and 1.14 kg/day, respectively.

**Table 4.** Influence of physical form of supplement, loose vs. pelleted, on supplement intake behavior of yearling cattle grazing dryland pastures

Item	Treatments^1^		SEM^2^	-values				
	Loose	Pelleted		TRT^3^	PD^4^	YR^5^	TRT×PD^6^	TRT×YR^7^
Intake, kg				<0.01	<0.01	0.80	<0.01	0.58
0 to 42 d	0.42	0.59	0.05					
42 to 84 d	0.93	1.35	0.05					
Intake, g/kg body weight				<0.01	<0.01	<0.01	<0.01	0.92
0 to 42 d	1.16	1.53	0.14					
42 to 84 d	2.19	3.22	0.14					
Intake rate, g/min				<0.01	<0.01	<0.01	0.10	<0.01
Year 1	80.4	226.6	6.90					
Year 2	79.8	137.5	7.03					
Time at supplement, min/d				0.12	<0.01	<0.01	0.03	0.24
0 to 42 d	10.10	7.13	0.90					
42 to 84 d	14.87	14.14	0.90					
Variation of supplement intake, %				0.20	0.03	0.03	0.28	0.56
0 to 42 d	137.4	99.9	13.4					
42 to 84 d	95.2	87.2	13.5					

Treatments are 1) Control, no supplement, 2) Supplement in loose form, 3) Supplement in pelleted form.

SEM = Standard Error (*N* = 20).

Treatment main effect (TRT).

Period main effect (PD); period 1: 0 to 42 d; period 2: 42 to 84 d.

Year main effect(YR).

Treatment × period interaction.

Treatment × year interaction.

There was a treatment × year interaction for heifer intake rate (*P* < 0.01) with heifers offered pelleted supplements consuming 2.8 and 1.7 times faster than heifers offered loose supplement in years 1 and 2, respectively. Additionally, intake rate was 22% lower (*P* < 0.01) in period 1 as compared to period 2 (115.0 ± 4.14 vs. 147.0 ± 4.01). A treatment × period interaction (*P* = 0.03) was observed for time spent at the supplement feeder with heifers fed pelleted supplements spending less time (*P* = 0.02) at the feeder during period 1 compared to heifers fed loose supplement. However, no difference (*P* = 0.57) was observed in period 2 with heifers spending on average 14.5 min per day at the supplement feeders. Time spent at the supplement feeder also displayed a year effect (*P* < 0.01), where heifers spent more time at the supplement feeders the second year of the study compared to the first year (9.03 ± 0.82 and 14.09 ± 0.82). Variation in supplement intake (% CV) was greater (*P* = 0.03) in period 1 compared to period 2, averaging 119% and 91%, respectively. In addition, variation in supplement intake was greater (*P* = 0.03) in year 2 than year 1, averaging 122% and 88 %.

## DISCUSSION

Ruminant production systems research continues to strive for precision management of nutrient use in grazing environments with strategic supplementation approaches that optimize the use of forage resources. In extensive rangeland environments, supplements are often provided in self-fed forms because of the difficult terrain, lack of accessibility to animals, and reduced labor requirements (Bowman and Sowell, 1997; Kunkle et al., 2000). Methods to limit intake of free-choice self-fed supplements often involve the use of salt combined with various changes in texture, supplement forms, bitterness, and hardness. These methods, however, often result in considerable intake variation and, as a result, reduced effectiveness of nutrient delivery.

This study focused on the intake and intake behavior of heifers consuming loose versus pelleted forms of the same supplement. Pelleting increased supplement intake by 24% to 32% as compared to the same supplement in a loose or granular form. The intake levels of this study were similar to those reported by Wyffels and coworkers (2020) in a winter grazing environment where cattle consumed a pelleted salt-limited supplement at 0.5 to 2.5 g/kg BW/d. Supplement intake rate (g/min) was 1.8 to 2.7 times greater with the pelleted supplement suggesting that the “intake-limiting” effects of salt were dramatically reduced in the pellet form. Since the majority of the oral cavity is filled by the tongue of the beef cow (Church, 1975; Cheeke and Dierenfeld, 2010), a loose-form supplement with more surface area most likely covers more gustatory, or taste bud receptors compared to a pelleted form. Therefore, the prehensile grasping of supplement by a heifer may result in larger bite sizes due to the hardened texture of a pelleted-form and less surface area contact with taste buds on the tongue. This assumes that the negative aversion to high salt is mediated through gustatory or taste bud receptors rather than digestive or metabolic effects of the increased salt (Goetcher and Church, 1970; Church, 1975; Cheeke and Dierenfeld, 2010).

Corresponding to large increases in supplement intake and intake rate; time spent at the supplement feeder was reduced early in the grazing period and did not differ in the latter portions of the grazing period for heifers consuming pelleted supplement compared to heifers consuming the loose form. Forage quality may have played a role in reducing the time spent at the supplement feeders early in period 1 due to the availability of higher quality forage. Time spent at the supplement feeders are surprisingly consistent ranging from 5 to 15 minutes per day and are similar to other studies using similar research technology but other supplement forms and forage conditions (Reuter et al., 2017; McClain et al., 2020; Wyffels et al., 2020). Therefore, the increase of supplement intake did not result in increased time at the supplement feeder allowing the animals equal access to grazing opportunities.

Variation of supplement intake (% CV) was not influenced by supplement form but declined in the later portions of the grazing season. However, variation of supplement intake ranged from 80% to 120 % of the mean intake. This observation is consistent with recent research evaluating salt-limited supplement intake (Reuter et al., 2017; Williams et al., 2018; Wyffels et al., 2020) as well as researchers evaluating baked molasses block intake which combines salt, texture and hardness intake limiter mechanisms (McClain et al., 2020; Parsons et al., 2021; Wyffels et al., 2021). The decrease in intake variation later in the grazing period has also been observed by other researchers (McClain et al., 2020; Wyffels et al., 2021).

Supplement intake increased and variation in supplement intake declined in the second half of the grazing period. While heifer gains for this study would be considered adequate over the 84-d grazing periods; forage quantity and quality was greatest at the beginning of the grazing period and declined to levels below the nutrient requirements of growing yearling heifers for the second half of the grazing period (NASEM, 2016). Similar to our study, other researchers have observed forage quality/quantity impacts on supplement intakes with intake increasing with declining forage quality and availability (Wagnon, 1965; Ducker et al., 1981; Bowman and Sowell, 1997). It has also been reported that the limiting effects of salt on supplement intake decline over time with ruminants increasing tolerance to high salt levels when fed for long periods (Kunkle et al., 2000).

## CONCLUSIONS

Our results suggest that salt-limited supplements have a high degree of overall intake variation including variation between animals, over time periods and across years. Physical form modification, such as pelleting, has a masking effect on the intake limiting influence of supplemental salt as indicated by the higher intake and intake rate of the pelleted supplement. Therefore, the physical form of supplement (loose vs. pelleted) should be considered in precision supplementation strategies. In addition, supplement intake increases over time with an increasing delivery of nutrients with declining forage quality and availability. This research contributes to the continued efforts to refine strategic supplementation practices that provide the right amount of nutrients, to the target animals, at the right time.
